# A comparison of the uniaxial deformation of copper and nickel (1 1 19) surfaces: a molecular dynamics study

**DOI:** 10.1038/srep42234

**Published:** 2017-02-07

**Authors:** Nuša Pukšič, Monika Jenko, Matjaž Godec, Paul J. McGuiness

**Affiliations:** 1 Institute of Metals and Technology, Physics and Chemistry of Materials, Ljubljana, 1000, Slovenia; 2 Jožef Stefan International Postgraduate School, Ljubljana, 1000, Slovenia

## Abstract

While a lot is known about the deformation of metallic surfaces from experiments, elasticity theory and simulations, this investigation represents the first molecular-dynamics-based simulation of uniaxial deformation for the vicinal surfaces in a comparison of copper and nickel. These vicinal surfaces are composed of terraces divided by equidistant, mono-atomic steps. The periodicity of vicinals makes them good candidates for the study of the surface steps’ influences on surface dynamics. The simulations of tensile and compressive uniaxial deformations were performed for the (1 1 19) vicinal surfaces. Since the steps on the surfaces serve as stress concentrators, the first defects were expected to nucleate here. In the case of copper, this was found to be the case. In the case of nickel, however, dislocations nucleated beneath the near-surface layer affected by the displacement field generated by the steps. Slip was hindered at the surface step by the vortex in the displacement field. The differences in the deformation mechanisms for the Ni(1 1 19) and Cu(1 1 19) surfaces can be linked to the differences in their displacement fields. This could lead to novel bottom-up approaches to the nanostructuring of surfaces using strain.

In the past 20 years investigations of stress and strain effects in the field of surface physics have become increasingly important as the manufacture of nanoscale materials and devices has become more widespread, leading to studies based on a combination of theoretical, computational and experimental investigations^1^. As a result, the definitions of surface stress and surface strain that allow for numerical thermodynamic studies based on atomistic models have now been established, and elastically mediated interactions between various surface defects such as “steps” and “islands” have been described. It is clear that the displacement fields induced by these surface defects can profoundly affect the morphology of the surface. For example, in homo- or hetero-epitaxial growth, steps on a surface can act as nucleation centres or decrease the diffusion of adsorbed species due to the Ehrlich–Schwoebel barrier; in catalytic reactions stepped surfaces often have a different catalytic activity to that of flat surfaces^2^,^3^,^4^,^5^,^6^,^7^. Controlled deformation also appears to be a potential tool for the self-organization of surfaces, applicable in a bottom-up approach to the nanostructuring of materials.

Elasticity also provides pressure maps that can help to predict the stress release resulting from atomic rearrangements^1^; however, on the subject of vicinal surfaces under an externally applied stress the data from both experiments and simulations is scarce. These vicinal surfaces are defined by terraces divided by equidistant, monoatomic steps, and can be exposed by cutting a crystal at a small angle to one of the low-index planes. The elastic fields that the steps induce in the crystal reach into the bulk and therefore influence the mechanical properties of the surface^1^,^2^,^8^,^9^,^10^,^11^.

Experiments on the nanoscale can be very time consuming and costly, requiring expensive equipment, particularly sensors; however, they confirm an array of unusual mechanical properties of nanoscale materials^12^. This serves as a motivation to use atomistic simulations in order to better understand the deformation mechanisms on the nanoscale. In previous studies of the mechanical properties of nanocrystalline materials and thin films by molecular statics and dynamics, most of the research was centered on the influence of phase and grain boundaries on the response of the material to an external load or deformation. And when molecular dynamics simulations of the plastic deformation of nanocrystalline materials are considered, the focus is mainly on the generation of dislocations at the grain boundaries and the effectiveness of the grain boundaries in slowing the rate of plastic deformation^13^,^14^,^15^,^16^,^17^.

Nano-indentation studies focus on dislocation nucleation beneath the indenter. The stress that is applied via the frictionless rigid indenter is non-uniform, but it can lead to homogeneous defect nucleation in a perfect crystal. The substrates are thin films^18^,^19^,^20^, with controlled impurities, i.e., vacancies^21^, voids^22^, grain boundaries^23^ and steps^24^,^25^,^26^,^27^, added in some cases.

As nanowires have a large surface-to-bulk ratio, the most comprehensive studies of the influence of free surfaces on the mechanical properties of metallic materials using molecular dynamics were made with samples of nanowires^28^,^29^,^30^,^31^,^32^. For instance, in nanowires, the orientation of the surface can influence which deformations are energetically favorable, overriding the crystallographically preferred deformation mechanisms^31^.

In this study molecular dynamics simulations of copper and nickel (1 1 19) vicinal surfaces under an externally applied uniaxial strain were performed and the beginning of plastic deformation was studied in order to determine the stability of the surface steps during deformation and their influence on the deformation mechanisms. The (1 1 19) surfaces were chosen because the steps on this surface, vicinal to the (1 0 0) nominal surface, are dense, and yet the distance between the steps is large enough to ensure that the interaction between the steps is weak: the relaxations of the step edge atoms are only slightly modified by the presence of the other steps^2^.

## Results

**Figure 1:**
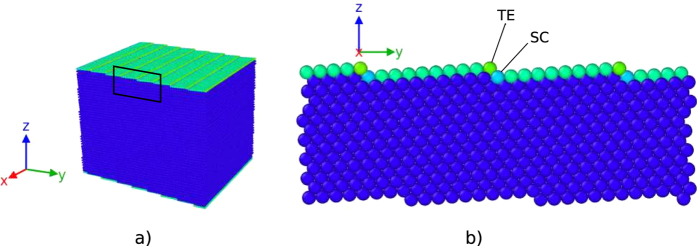
Two-dimensional schematic of a (1 1 19) surface. The terrace edge (TE) and step corner (SC) atoms are marked. The width of the terraces is 10 atomic rows. Orientation of the terraces is (001). The (1 1 19) and (19 19 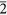) directions correspond to the **z** and **y** simulation box axes. The **y** direction is also the direction of the applied strain.

A two-dimensional schematic of a (1 1 19) surface is shown in Fig. 1. These vicinal surfaces are composed of (001)-oriented terraces divided by equidistant, monoatomic steps with (111) microfacets. There are 10 rows of atoms per terrace. The terrace edge (TE) and step corner (SC) atoms are marked.

### Displacement fields

The steps on the surface of a metallic material play a role in the atomic arrangement of the surface layers. Near-surface atoms are displaced from their bulk positions to accommodate the lack of neighbours at the surface. In the case of the vicinal surfaces this minimal energy arrangement of atoms leads to a complex displacement field, stretching several layers into the bulk specific for each surface orientation and material^2^,^8^.

Although the displacement fields have been studied for a range of metals and surface orientations, their influence on the deformation mechanisms of the surfaces has never been investigated before. We have compared the displacement fields of Ni(1 1 19) and Cu(1 1 19) surfaces with their deformation mechanisms to investigate whether any influence of the displacement fields on the deformation mechanisms could be discerned.

**Figure 2:**
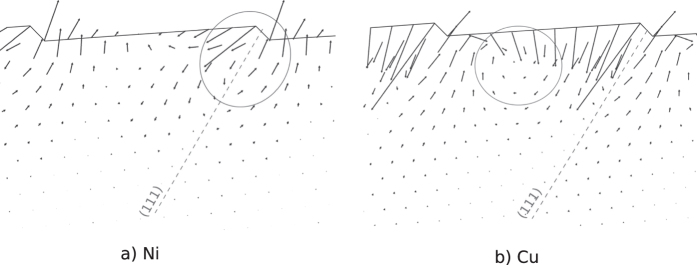
Displacement fields of Ni(1 1 19) and Cu(1 1 19) surfaces are shown. Arrows show the displacements of the atoms from their bulk positions (multiplied by 100 for clarity). Surface atoms are traced by grey lines. Dashed lines mark the (111) planes. The general regions of the displacement vortices are circled. In the case of the Ni(1 1 19) surface (**a**) some of the terrace atoms are displaced towards the bulk and others away from the bulk. The step corner and terrace edge atoms are displaced the most and the atoms in the middle of the terrace are displaced the least. The vortex is centered on the (111) plane marked with a dashed line. In the case of the Cu(1 1 19) surface (**b**) all the terrace atoms are displaced towards the bulk, whereas the step corner atom is displaced outwards. The displacements form a vortex underneath the terrace.

The displacement fields for the Ni(1 1 19) and Cu(1 1 19) surfaces are shown in Fig. 2. Dots denote the atoms and arrows show their displacements from the bulk positions (multiplied by 100 for clarity). The grey lines trace the surface atoms. The step height is slightly reduced by the displacements, as TE atoms are displaced towards the bulk and SC atoms are displaced outwards.

In the case of nickel (Fig. 2a), some of the terrace atoms are displaced towards the bulk and some away from the bulk, with a turning point in the middle of the terraces, where the displacements are minimal. The TE and SC atoms are displaced the most. The magnitudes of the displacements of the TE and SC atoms are 0.007 nm and 0.005 nm, which equal 1.9% and 1.3% of the lattice parameter, respectively. The displacements form vortices that are located near the steps.

In the case of copper (Fig. 2b), all the terrace atoms are displaced towards the bulk and the step corner atoms are displaced outwards. The TE and SC atoms are displaced the most. The magnitudes of the displacements of the TE and SC atoms are 0.013 nm and 0.004 nm, which equal 3.6% and 1.2% of the lattice parameter, respectively. The displacements form vortices that are located underneath the terraces.

In the case of nickel, the vortices can be thought of as straddling the (111) plane intersecting with the step, whereas in the case of copper the vortices can be seen as contained between two (111) planes intersecting with adjacent steps. In the view presented in Fig. 2, the vortices in nickel are rotated counter-clockwise and the vortices in copper clockwise.

### Results of deformation simulations

Copper and nickel both have a face-centered cubic (fcc) lattice. In fcc crystals the close-packed planes and directions are preferred for slip. However, which slip systems are the most favorable is additionally determined by the direction of the strain. In our case the strain direction was (19 19 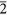); therefore, the primary slip systems are {111} 〈0
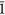
1〉 and {111} 〈
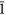
01〉.

As both materials have low stacking-fault energy, both are expected to deform by partial dislocations and twinning.

#### Stress-strain curves

**Figure 3:**
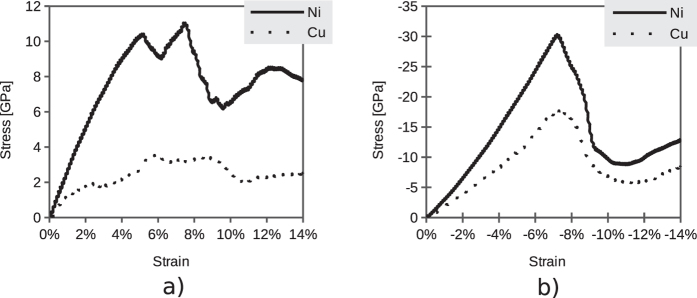
Stress-strain curves for Ni and Cu(1 1 19) oriented thin films deformed in tension and in compression. Both materials exhibit a yield-stress asymmetry. (**a**) In the case of Ni, the peaks at 5.5% and 7.5% strains indicate the activation of the first and the second slip system. For copper, the peak at 6.0% strain indicates the activation of the first slip system, and the peak at 9.3% strain the beginning of twinning. (**b**) In the case of compression, multiple deformation mechanisms are activated at once at −7.2% strain for nickel and at −7.1% strain for copper.

The stress-strain curves for tensile deformation are shown in Fig. 3a, and those for compression are shown in Fig. 3b. Both materials exhibit a significant yield-stress asymmetry, with the yield stress being higher in compression than in tension. Both materials exhibit multiple peaks in the stress-strain curves in tensile deformation, Fig. 3a, and a single pronounced peak in compressive deformation, Fig. 3b. For nickel in tension, the peaks at 5.5% and 7.5% strains indicate the activation of the first and the second slip system. For copper in tension, the peak at 6.0% strain indicates the activation of the first slip system, and the peak at 9.3% strain the beginning of the twinning. In the case of compression, multiple deformation mechanisms are activated at once at −7.2% strain for nickel and at −7.1% strain for copper, leading to a larger and steeper drop in stress compared to the case of tensile deformation.

#### Tensile deformation

**Figure 4:**
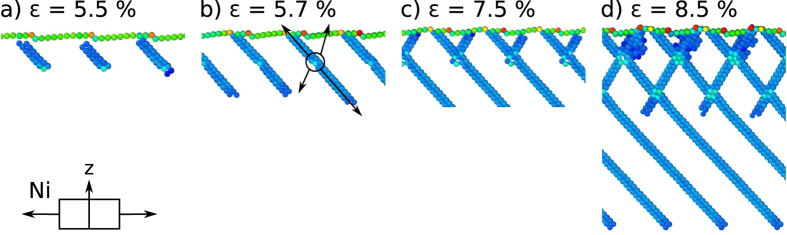
Deformation evolution of Ni(1 1 19) deformed in tension. (**a**) Dislocations nucleate in the bulk 8–10 layers underneath the surface. (**b**) The active slip system is perpendicular to the one expected and leads to the formation of additional steps on the surface. (**c**) After the stacking faults reach the bottom of the sample, the next slip system is activated. Partial dislocations nucleate at the same nucleation sites in the bulk, grow towards the surface and then into the bulk. (**d**) At strains above 8.0% a set of stacking faults begins to grow into twins. Atoms comprising surfaces and stacking faults are shown, others (*CS* < 2) are removed for clarity.

In the case of Ni, Fig. 4, the first slip system is activated at the strain of 5.5%, when dislocations nucleate in the bulk, 8–10 layers below the surface, Fig. 4a. Stacking faults first climb towards the surface, where they intersect with the terraces and not with the steps. Slip introduces new steps on the surface, Fig. 4b. Stacking faults then continue growing into the bulk of the sample by dislocation climb in the opposite direction in the same slip plane. When the stacking faults reach the layer of fixed atoms at the bottom of the sample, the slip system with the perpendicular slip direction is activated, at the strain of 7.5%, Fig. 4c. These stacking faults originate at the same nucleation sites as the previous set. Another set of steps is introduced at the surface and a crosswise pattern of stacking faults emerges in the bulk, Fig. 4d. In addition, at strains above 8.0% a set of near-surface stacking faults grow into twins.

**Figure 5:**
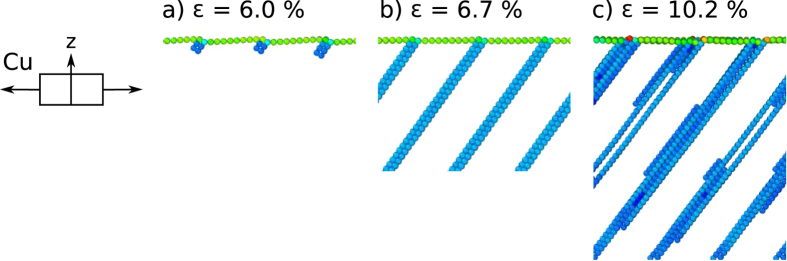
Deformation evolution of Cu(1 1 19) deformed in tension. (**a**) Stacking faults nucleate at the surface, (**b**) growing to the bottom of the sample. (**c**) The perpendicular slip system is not activated, instead deformation mechanism switches to twinning. Atoms comprising surfaces and stacking faults are shown, others (*CS* < 2) are removed for clarity.

In the case of Cu, Fig. 5, Shockley partial dislocations nucleate at the steps on the surface and climb into the bulk, creating stacking faults reaching from the surface ever deeper into the bulk with increasing strain, Fig. 5a,b. Slip reduces the height of the steps and leads to a flattening-out of the surface. No trailing partials detach from the surface. When the stacking faults reach the bottom of the sample, Fig. 5c, stacking faults grow into twins.

In comparison, dislocations nucleate at the surface steps in copper and in the bulk in nickel. The steps on the surface act as stress concentrators and are the expected nucleation sites. In both metals the dislocations nucleate and travel evenly along the whole length of the simulated samples in the **x** direction, parallel to the direction of the steps. In addition, the primary slip system in copper is the one expected to be activated in tensile deformation, leading to a flattening-out of the surface. In the case of nickel the primary slip system is perpendicular to the one expected and leads to the formation of additional steps on the surface. Both materials start twinning at strains above 8.0%.

#### Compressive deformation

In the case of nickel, dislocations nucleate at the steps and in the bulk 8–10 layers beneath the surface (a). The bulk nucleation sites in nickel are the same as in the case of tensile deformation. Two perpendicular slip systems are activated at once. A dense mesh of dislocations forms. Due to the lack of space available, the dislocations that have nucleated at the surface climb along the terraces instead of growing into the bulk. In the case of copper, only one slip system is activated (b). As expected, the slip plane is perpendicular to the one active in tensile deformation. After the dislocations reach the bottom of the sample, deformation twinning is observed in the bulk (d). The slip system perpendicular to the primary one is not activated. In comparison, the bulk nucleation sites are present in nickel but not in copper and two slip systems are active in nickel, whereas only one is active in copper. In addition, deformation twinning is observed in copper but not in nickel.

**Figure 6:**
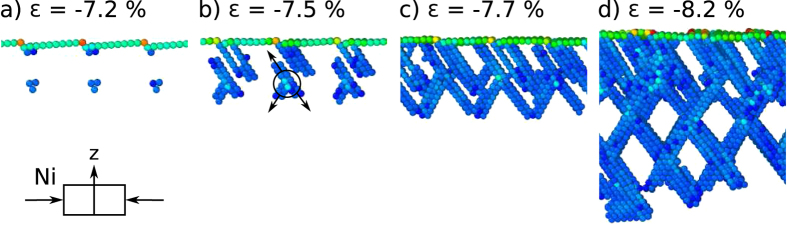
Deformation evolution of Ni(1 1 19) deformed in compression. (**a**) Dislocations nucleate in the bulk 8–10 layers underneath the surface as well as at the steps. (**b**) From the nucleation sites in the bulk, stacking faults grow in three directions, utilizing two slip planes. Stacking faults originating at the steps switch to twinning at the strain of −7.3%. (**c**) Stacking faults and twins connect. (**d**) Defects grow into the bulk evenly along both active slip systems. Atoms comprising surfaces and stacking faults are shown, others (*CS* < 2) are removed for clarity.

In the case of nickel deformed in compression, Fig. 6, dislocations nucleate at the surface and in the bulk, Fig. 6a. The deformation mechanism is a combination of slip and twinning. One set of partial dislocations nucleates at the edges of the terraces. Hindered by the dislocations nucleating in the bulk, these partial dislocations switch to twinning instead of climbing deeper into the bulk, Fig. 6b. The partial dislocations nucleating in the bulk, 8–10 layers below the surface, grow along three slip directions on two slip planes, Fig. 6b. The placement of the nucleation sites is the same as in the case of nickel deformed in tension. A complex crosswise pattern of dislocations and twins emerges, Fig. 6c, and continues to grow evenly into the bulk along both active slip systems, Fig. 6d. Compared to tensile deformation, where twinning emerges at strains higher than 8.0%, in tension twinning emerges sooner at a strain of −7.5%.

**Figure 7:**
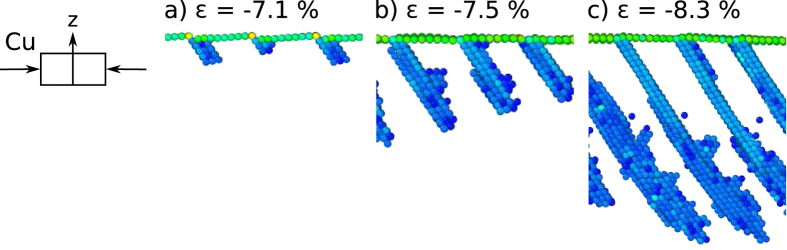
Deformation evolution of Cu(1 1 19) deformed in compression. (**a**) Defects nucleate at the steps on the surface. (**b**) Parallel twins grow into the bulk along the direction of the active slip system. (**c**) Twins remain the prevailing deformation mechanism, the perpendicular slip system is not activated. Atoms comprising surfaces and stacking faults are shown, others (*CS* < 2) are removed for clarity.

In the case of copper deformed in compression, dislocations nucleate at the edges of the terraces, Fig. 7. The deformation mechanism is a combination of slip and twinning. The slip direction is perpendicular to the one active in tensile deformation. Slip again flattens out the surface. Trailing partial dislocations nucleate at the surface in some places. The slip system perpendicular to the primary one is not activated in this case. Twinning is the prevailing deformation mechanism.

In comparison, the bulk nucleation sites are observed in nickel, but not in copper. Two perpendicular slip system are active in nickel, while only one is active in copper. Deformation twinning occurs in both materials, starting at strains of −7.1% for copper and −7.5% for nickel.

## Discussion

We have investigated the plastic deformation of the (1 1 19) vicinal surfaces of copper and nickel thin films deformed in tension and in compression with a uniaxial engineering strain at a rate of 2.5% ps^−1^. Although the metals are very similar, we have found very significant differences in their plastic deformation mechanisms.

The low temperature and the high strain rate of the simulations influence the deformation mechanisms. At low temperatures the diffusion-controlled processes are suppressed and the main deformation mechanism is slip^33^. At high strain rates the dislocation mobility is lowered, giving precedence to dislocation generation^33^. Low temperatures and high strain rates can also lead the deformation mechanisms to transition from slip to deformation twinning^33^.

In the size range of our simulations, i.e., approximately 10 nm, partial dislocations are generally preferred over full dislocations as the deformation mechanisms for metallic materials^12^,^34^.

As both metals have low stacking-fault energies, the expected deformation mechanisms were by partial dislocation slip and by twinning. As the stacking fault energy and the predicted twinning stress of nickel are higher than that of copper, a preference of twinning over slip is expected to be more pronounced in copper^35^. We found that to be the case in tension and in compression. In addition, nickel is able to endure exceptional elastic lattice distortion^36^.

Multiple criteria are met, for both materials, predicting partial dislocations and twinning as the main deformation mechanisms, and both are observed in all the cases, superseding full dislocation nucleation.

The arrays of steps on the (1 1 19) vicinal surfaces influence the deformation mechanisms of both metals. The regularity of the steps is translated into the regularity of the stacking faults that form in all cases.

In the case of copper, steps on the surface serve as nucleation points and both tensile and compressive strains lead to a flattening out of the surface steps. This is as expected from the theory of elasticity.

In the case of nickel, the point of dislocation nucleation lies 8–10 layers underneath the surface instead of at the surface. The nucleation sites are the same in tensile and compressive deformation, although in compression the dislocations nucleate at the surface steps as well. The defect nucleation sites buried below the surface are unexpected, since the steps are known as the stress-concentration points. The defect nucleation sites lie beneath the terraces just outside the region affected by the displacement field. In addition, the primary slip system activated in nickel deformed in tension is perpendicular to the primary system activated in copper.

In the case of copper deformed in tension, slip occurs in the (111) plane that delineates two vortices underneath adjacent terraces. The atoms involved slip in the direction of their displacements. In the case of nickel, this process is hindered by the presence of the vortices. Nucleation points are shifted into the bulk beneath the displacement field. Slip occurs in the direction in which crystal symmetry is disturbed the most by the displacement field.

In the case of nickel deformed in compression, nucleation points in the bulk and at the steps are activated. The area of the vortex is again initially avoided, then cut through by the stacking fault originating in the bulk, as was the case in tensile deformation. The nucleation of stacking faults at the steps is in this case not restricted by the displacement field.

Zimmerman *et al*.^24^ studied Au(111) surfaces with single monoatomic steps at 0 K and found that the step had no long-range influence on the deformation mechanism, and only when the indenter came into physical contact with the step, did the deformation mechanism beneath the indenter switch from dislocation emission from the surface to slip aligned with the step. We also found a preference for slip aligned with the steps.

Shan *et al*.^25^ performed a similar study of nano-indentation on thin copper films and also found no long-range effects of the step. In addition, they found that in the case when the indenter came into contact with the step, there was a significant difference in the strength of the first dislocation emission. Apart from that, it was not made clear whether any change in the deformation mechanism was observed.

Lu *et al*.^26^,^27^ investigated the effects of surface steps on nano-indentation in Al. They found that the shear stress resulting from the step influences the choice of the active slip systems and lowers the threshold for plastic deformation.

To date no such investigations were performed for nickel vicinal surfaces.

As the surfaces were first to deform in our study, we are in agreement with Shan *et al*.^25^ and Lu *et al*.^26^,^27^, that the steps influence the choice of the active slip system and have a lower threshold for plastic deformation compared to the bulk.

We have observed distinct deformation mechanisms depending on the sign of the strain. Both materials were similarly affected, as a switch to a different deformation mechanism occurred in tensile deformation at higher strains, while multiple deformation mechanisms were activated at once in compression. Experiments show there are cases of different deformation mechanisms depending on the sign of strain^37^ as well as switching of deformation mechanisms at higher strains^37^.

## Conclusions

We conclude that the displacement fields of vicinal surfaces play a role in their deformation mechanisms. As expected, the regularity of the steps on the surface translates into the regularity of the dislocations and stacking faults that form beneath the surface. While all the dislocations were expected to nucleate at the steps, this was the case for copper, but not for nickel. In tensile deformation, the displacement field in the vicinity of the step in nickel seems to prevent slip in the (111) plane, which is the first to activate in the case of copper. In compressive deformation, the nucleation points beneath the surface in nickel are activated in addition to the ones at the surface that follow the expected pattern, as evidenced in copper.

As the displacement fields are orientation dependent, differences in the deformation mechanism might also arise for other vicinal surfaces.

As was shown for nickel, a patterned surface can be achieved, which has repercussions for bottom-up approaches to the nano-structuring of surfaces using strain. A high density of steps can be achieved, which could be used for templates, in catalysis^3^,^4^,^5^,^6^,^7^,^38^ or for orienting or growing nanotubes or nanowires^39^,^40^.

## Methods

Copper and nickel thin films of 10 nm thickness with (1 1 19) surface orientation were simulated. The simulations of the tensile and compressive uniaxial deformations were performed at 10 K, applying engineering strain at a strain rate of ±2.5% ps^−1^. The strain direction was (19 19 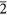), perpendicular to the steps.

Copper and nickel single-crystal substrates were used in the molecular dynamics simulations, which were carried out using the LAMMPS Molecular Dynamics Simulator^41^ and the following EAM potentials: for copper by Mishin *et al*.^42^ and for nickel by Sheng *et al*.^43^.

The axes of the simulation boxes were **x** = (1 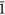 0), **y** = (19 19 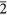) and **z** = (1 1 19).

The boundaries of the simulation boxes were periodic in the **x** and **y** directions and fixed in the **z** direction.

In the **y** direction the size of the simulation box was subject to requirements for periodicity of the terraces and steps for vicinal surfaces. The number of steps and terraces included was 8.

In the **z** direction the simulated films were bound by a free surface at the top and by a layer of fixed atoms at the bottom to prevent the loss of atoms at the border of the simulation box and to simulate defect-free bulk. The thickness of this frozen layer was 1.2 nm. These atoms were fixed at the positions they had when equilibrated to 10 K and were remapped at every deformation of the simulation box. All the forces acting on these atoms were otherwise reset to zero.

Physical dimensions of the simulated samples were as follows. The lattice parameter of copper was 0.36149 nm and the size of the simulated sample was 20.4 nm × 19.5 nm × 11.2 nm. The lattice parameter of nickel was 0.35200 nm and the size of the simulated sample was 19.9 nm × 19.0 nm × 11.2 nm.

Damped dynamics^44^ were used to find the minimum energy states of the surfaces at 0 K. Afterwards, the substrates were equilibrated to 10 K. Then, to simulate engineering compressive and tensile strains, the simulation box was deformed in the **y** direction in increments of 0.5% with 200 fs periods of equilibration in-between, resulting in the overall strain rate of 2.5% ps^−1^. The stress in the **x** direction was kept to zero.

OVITO^45^ was used for visualization and analysis of the simulation data.

The centro-symmetry parameter is used to identify dislocations and stacking faults in the simulations. In solid-state systems the centro-symmetry parameter is a useful measure of the local lattice disorder around an atom and can be used to characterize whether the atom is part of a perfect lattice, a local defect (e.g., a dislocation or stacking fault), or at a surface. This parameter is computed using the following formula^46^


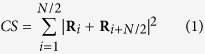


where the *N* nearest neighbours of each atom are identified and **R**_*i*_ and **R**_*i*+*N*/2_ are vectors from the central atom to a particular pair of nearest neighbors. For an fcc lattice *N* is 12. For an atom on a lattice site, surrounded by atoms on a perfect lattice, the centro-symmetry parameter will be 0. It will be near 0 for small thermal perturbations of a perfect lattice. If a point defect exists, the symmetry is broken, and the parameter will be a larger positive value. An atom at a surface will have a large positive parameter.

Primary slip systems are defined as having the largest Schmid factors for the given strain direction, which are calculated as *m* = (**s**_**P**_ · **y**) (**s**_**D**_ · **y**), where **s**_**P**_, **s**_**D**_ and **y** denote the slip-plane normal, the slip direction and the strain direction.

## Additional Information

**How to cite this article**: Pukšič, N. *et al*. A comparison of the uniaxial deformation of copper and nickel (1 1 19) surfaces: a molecular dynamics study. *Sci. Rep.* **7**, 42234; doi: 10.1038/srep42234 (2017).

**Publisher's note:** Springer Nature remains neutral with regard to jurisdictional claims in published maps and institutional affiliations.
